# Not all cicadas increase thermal tolerance in response to a temperature gradient in metropolitan Seoul

**DOI:** 10.1038/s41598-020-58276-0

**Published:** 2020-01-28

**Authors:** Hoa Quynh Nguyen, Hortense Serret, Yoonhyuk Bae, Seongmin Ji, Soyeon Chae, Ye Inn Kim, Jeongjoo Ha, Yikweon Jang

**Affiliations:** 1 Interdisciplinary Program of EcoCreative, Ewha Womans University, Ewhayeodaegil-52, Seoul, 03760 Republic of Korea; 2 Department of Life Science and Division of EcoScience, Ewha Womans University, Ewhayeodaegil-52, Seoul, 03760 Republic of Korea; 3 Department of Life Science, Hallym University, Hallimdaehakgil-1, Chuncheon, 24252 Republic of Korea; 4 Division of Life Sciences, Korea University, Anamro-145, Seoul, 02841 Republic of Korea

**Keywords:** Behavioural ecology, Ecophysiology

## Abstract

Rapid anthropogenic alterations caused by urbanization are increasing temperatures in urban cores, a phenomenon known as the urban heat island (UHI) effect. Two cicada species, *Cryptotympana atrata* and *Hyalessa fuscata* (Hemiptera: Cicadidae), are abundant in metropolitan Seoul where their population densities correlate strongly with UHI intensities. Such a positive correlation between cicada density and UHI intensity may be possible if cicada abundance is linked to a certain thermal tolerance. We tested this hypothesis by investigating variation in morphology and thermal responses of two cicada species along a thermal gradient in Seoul and surrounding areas. The morphological responses were measured by metrics such as length, thorax width and depth, and mass. The thermal responses were measured in terms of minimum flight temperature, maximum voluntary temperature and heat torpor temperature. First, we observed a species-specific variation in thermal responses, in which *C. atrata* displayed a higher thermal threshold for maximum voluntary and heat torpor temperatures than *H. fuscata*. Second, a positive association between temperature conditions and body sizes were displayed in females *H. fuscata*, but not in either conspecific males or *C. atrata* individuals. Third, *C. atrata* exhibited similar thermal responses regardless of habitat temperature, while *H. fuscata* in warmer areas showed an increase in heat tolerance. In addition, *H. fuscata* individuals with bigger thorax sizes were more heat-tolerant than those with smaller thoraxes. Overall, our research is the first to detect a variation in thermal responses and body size of *H. fuscata* individuals at a local scale. More investigations would be needed to better understand the adaptation mechanisms of insects linked to UHI effects.

## Introduction

Temperature is one of the most important abiotic factors affecting daily activities and the life history of cicadas^1–6^. As temperature directly dictates chemical rates and metabolic processes *in vivo*, a habitat with fluctuating temperatures could drive insects to manage the high energy costs of maintaining body temperature, *T*_b_, within a certain range to coordinate reproductive activity^4^. The surrounding conditions may also have an effect on reproductive success and mortality^7–10^. If so, cicadas should select favorable thermal conditions to obtain optimal body temperatures via behavioral- or physiological mechanisms^4,11^.

The urban heat island (UHI) effect, in which increased temperatures are associated with urban areas, is a ubiquitous consequence of microclimatic perturbation^12^ due to human activities and energy consumption, along with modification of landscapes and urban geometry^13–17^. Elevated temperatures in urban environments are regarded as detrimental as they reduce plant photosynthesis capability^18^ and biological diversity^19,20^. Nevertheless, for urban herbivorous insects such as cicadas, such warm habitats may prove advantageous. First, post-morphogenesis development of cicada eggs require a large degree of thermal accumulation^2^. Second, growth rates of cicada nymphs demonstrate a positive association with climate: those inhabiting warmer regions are more likely to grow faster than counterparts in cooler regions^21^. This may facilitate the development of larger body size in cicadas in the former group^22^, which in turn directly promotes greater fecundity of females^23^ and indirectly contributes to higher mating success in males^24^. Third, the emergence of final instars is stimulated by soil temperature, with warmer conditions triggering an earlier phenology of those instars^25^. Such earlier eclosion of male over female cicadas^6^ works advantageously for males as it maximizes mating success in those multiple copulating individuals^26^.

Recent studies have shown that some urban insect species adapt rapidly to warm city cores by increasing thermal tolerance. Herbivorous insects with high thermal tolerance capacity are better able to adapt to urban environments^27^. Specific physiological tolerances and thermoregulating behaviors of insects vary with their physical habitat conditions^28^. Accumulating evidence of an ability to track localized thermal profile clines in cities suggests that urban insects can evolve adaptive traits in response to such rapid environmental changes and become overrepresented in cities^29,30^. In several forest ant species in North America, higher critical thermal maxima correlate positively with population abundances in warm urban plots^31,32^. Additionally, urban ants possess higher heat tolerance than do rural ants^33,34^, which indicates adaptive plasticity or local thermal adaptation to microclimatic change in urban organisms.

Two cicada species, *Cryptotympana atrata* and *Hyalessa fuscata* (Hemiptera: Cicadidae), are widely distributed on the Korean Peninsula. These cicadas emerge annually from June to early October for key life history events, such as mate attraction, pair formation, and oviposition. The distribution of *C. atrata* ranges from East Asia to the northern part of Indo-China, whereas that of *H. fuscata* overlaps East Asia and encompasses the Far East of Russia^35^. An enumeration survey of cicada exuviae depicts more prevalent population densities in urban and suburban habitats relative to countryside habitats^36^. In particular, they constitute a major portion of cicada species in metropolitan Seoul, where their population densities correlate highly with UHI intensities^37^. *C. facialis*, a closely related species to *C. atrata*, has shown rapid population expansion in urbanized areas in Japan, owing to superior thermal adaptation to urban conditions^2^. Provided that the prevalence of *C. atrata* and *H. fuscata* populations in urban area is due to greater thermal tolerance to urban conditions, their thermal responses can be expected to be associated with the localized clines of habitat temperatures intensities.

Our aim was to investigate variations in morphology and thermal responses of two cicada species, *C. atrata* and *H. fuscata*, in accordance with the temperature of their habitats. We collected cicadas along a thermal gradient and assessed their morphometry and thermal responses. Given that a positive correlation already exists between cicada population densities and ambient temperatures, we predicted positive associations between UHI intensities and (1) morphological characteristics and (2) thermal responses of those cicadas.

## Methodology

### Sample collection

We sampled populations of *C. atrata* and *H. fuscata* from July 15 to August 5, 2016, in metropolitan Seoul and the vicinity in the Republic of Korea. Metropolitan Seoul covers more than 600 km^2^ with diverse landscape features, generating a mosaic of heterogeneous UHI intensities for cicadas. Approximately 10 million people live within city limits, with another 10 million in the surrounding suburban areas.

**Figure 1:**
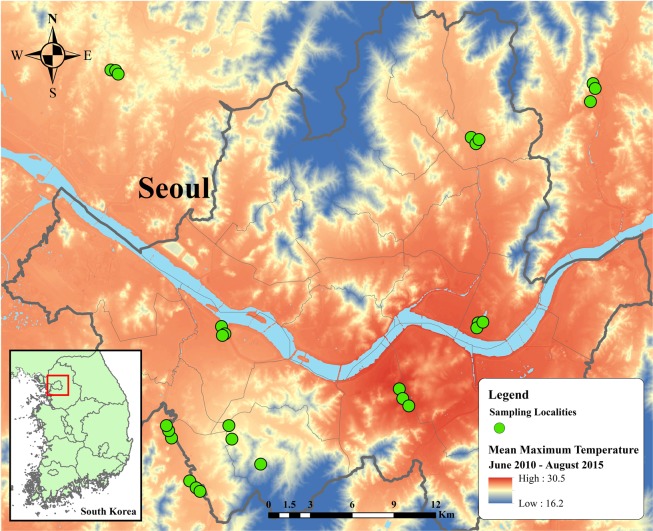
Distribution of sampling areas within and surrounding metropolitan Seoul. Thermal map of average maximum temperature from June 1, 2010, to August 31, 2015, in metropolitan Seoul constructed by ArcMap 10.5. Temperature ranges from 16.2 to 30.5 °C, indicated by dark blue to red regions. Green circles designate sampling localities. Inset map in the left bottom corner illustrates the regional location of metropolitan Seoul in the Republic of Korea.

The sampling method and selection of study areas followed 12 sampling areas by Nguyen, *et al*.^37^. From there, three areas were excluded due to low sampling densities and difficulty in collecting cicadas. In order to verify our sampling design and its suitability to our research question, we compared those nine areas in terms of the abiotic factors related to urbanization such as greenness, wetness, imperviousness and isothermal. Greenness is a measure of photosynthetically active vegetation, wetness represents soil and vegetation moisture^38^, and imperviousness measures building footprints, pavements and asphalt. Isothermal is mean diurnal range divided by temperature annual range, extracted from worldclim 1.4^39^. A one-way analysis of variance in which greenness, wetness, imperviousness, and isothermal were response variables was conducted. As expected, isothermal was the only variable that was significantly different among the nine areas (Supplementary Material 1). We concluded that temperature was the critical factor that differentiated among those nine area, thus verifying our sampling scheme (Fig. 1).

Weather data were compiled from Korea Meteorological Administration records. We calculated average maximum ambient temperature (*T*_a_Max) of each area during summer periods from June 1, 2010, to August 31, 2015. Given that mean, minimum, and maximum ambient temperatures are employed in UHI studies^40^, we relied on maximum ambient temperature, as it better represents the actual high ambient temperature that the cicadas encounter in their environment than the mean or minimum ambient temperature. Nine areas were randomly sampled and each area was sampled twice. Cicadas were collected from 8:00 a.m. to 12:00 p.m. at residential complexes in each area, and were subjected to thermal-response experiments within the day of capture. Information regarding sampling areas and the number of each species collected at each area are provided in Supplementary Material 2.

### Measurement of thermal responses

Minimum flight temperature (MFT), maximum voluntary temperature (MVT), and heat torpor temperature (HTT) are conservative measures of thermal adaptation of cicadas to a habitat^4,41,42^ (see^43^ for a summary of thermal responses in 118 taxa of North American cicadas). MFT represents the lowest body temperature with fully coordinated activity. MVT is the upper thermoregulatory point at which thermoregulation takes precedence over other behaviors. HTT is the upper limit beyond which cicadas sink into a state of torpor. MVT increases as a habitat becomes warmer in some cases, whereas HTT is strictly related to the thermal condition of a habitat^4,41–44^. The MFT-to-HTT range indicates the fully active thermal breadth (*T*_b_Range) of each species in relation to a certain habitat condition.

Thermal responses of each individual were assessed in a single assay with no rest time between treatments under laboratory conditions. First, each cicada was cooled to a torpid state by keeping it at −20 °C. We checked each individual’s *T*_b_ every three minutes to ensure they did not freeze, as this might affect HTT^42^. As soon as the individual was torpid, we assessed MFT by dropping the insect from a height of 2.5 m. If the insect could not perform the expected behavior, it was allowed to gradually warm up at ambient temperature for one minute before being reexamined. After that, the insect was placed under a heat lamp to obtain MVT. The *T*_b_ at which the individual moved away from the heat source and started to seek shade was determined to be its MVT. The cicada was continuously heated under the heat lamp until no movement was observed, at which point its *T*_b_ indicated HTT. The procedure was not lethal, as individuals could recover to normal active conditions after several minutes. We acknowledged that this assay may have induced stress to some extent in cicadas. However, such measurement of thermal responses have been performed over several decades^43^, and further investigation is necessary to assess tentative influences of this assay on the thermal performance of cicadas.

The evaluation of *T*_b_ in cicadas is commonly conducted inside the mesothorax^43^. Here, we chose to assess *T*_b_ from both the mesothorax and the pronotum. Indeed, pronotom may represent an evaporative cooling site^45^. Evaporative cooling is a key physiological thermoregulation mechanism in cicadas, as it provides a major cooling effect by dissipating excess heat^45–47^ and facilitates the cicadas’ endurance of high ambient temperatures^4^. The temperature of the pronotum therefore may signal a critical thermal threshold for the individual to regulate *T*_b_ within its functionally active range and adopt necessary thermoregulation strategies to prevent excessive increase in *T*_b_.

All temperature measurements were performed using a digital thermometer with a k-type thermocouple (Omega; model #: 450–AKT; Norwalk, Connecticut, USA) sensitive to ± 0.25 °C. The total live body mass of each individual was determined using an Adventurer Pro Analytical (Ohaus; New York, USA) scale sensitive to ± 0.0001 g. We also measured body length, mesothorax width, and mesothorax depth using Digital Calipers (Insize Co., Ltd.; Georgia, USA) sensitive to ± 0.02 mm.

### Statistical analysis

#### Comparative thermal responses of *C. atratra* and *H. fuscata*

A first constrained multivariate analysis, i.e., redundancy analysis^48^ (RDA), was performed to compare thermal responses of MFT, MVT, HTT and *T*_b_Range between *C. atrata* and *H. fuscata*. Analyses were conducted separately for temperature measurements from the pronotum and mesonotum. *C. atrata* and *H. fuscata* responded differently to heat experiments. Therefore, we conducted hereafter analyses separately for each species.

#### Variation in morphological characters of each cicada species

First, we applied an RDA to evaluate the responses of the morphological characteristics measured by total mass (mass), body length (length), mesothorax width (width), and mesothorax depth (depth) to sex and *T*_a_Max. Second, intersexual morphological differences were assessed by performing *t*-tests for normally distributed data of mass and Kruskal-Wallis tests for non-normally distributed data of length, width and depth. Furthermore, we also conducted linear regressions to examine the effect of temperature on each of the morphological characteristics, separating analysis for males and females. We examined the assumption of homogeneity of variance of residuals of each linear regression model via a diagnostic plot of predicted values versus standardized residuals.

#### Variation in thermal responses of each cicada species

A third RDA was used to assess the thermal responses of MFT, MVT, HTT, and *T*_b_Range by sex, *T*_a_Max and width. We performed independent RDAs for temperature measurement from the pronotum and mesonotum. Again, we compared intersexual differences in terms of thermal responses, employing *t*-tests for normally distributed data (MVT, HTT and TB) and Kruskal-Wallis tests for non-normally distributed data (MFT). Linear regressions tested how both male and female thermal responses, measured from pronotum and mesonotum, were influenced by *T*_a_Max and width. Diagnostic plots of predicted values versus standardized residuals were visualized to assess the assumption of homoscedasticity of residuals for each linear regression model. RDA results showed no significant thermal responses of *C. atrata* in the measurement from the pronotum or mesonotum, and it was exempted from follow-up intersexual variation tests.

#### Intersexual variation in thermal responses of *H. fuscata*

Finally, an RDA was performed to quantify intersexual differences in thermal responses according to *T*_a_Max and width. The Vegan package^49^ on R Studio (Version 1.0.143) was used for all multivariate analyses, and the statistical significance of the entire model for each variable (marginal test) was evaluated using Monte-Carlo permutation tests (n = 999). Linear regressions were performed with SPSS 22 (IBM Corp.; New York, USA). Results are presented as the mean ± standard deviation.

### Ethics declaration

Cicadas are common species in Republic of Korea. Neither *C. atrata* nor *H. fuscata* was listed as protected or endangered species in the “List of wildlife species prohibited for collection” issued by the Ministry of Environment, Republic of Korea and in the IUCN Red List. Therefore, no field permit was required for this study.

## Results

### Comparative thermal responses of *C. atratra* and *H. fuscata*

Thermal responses measured at the pronotum (*n* = 158) showed that *C. atrata* became fully coordinated at an MFT of 24.81 ± 1.72 °C, reaching the thermoregulation level at an MVT of 37.58 ± 2.01 °C, and becoming heat torpid at an HTT of 46.92 ± 2.71 °C. Body temperatures measured inside the mesonotum (*n* = 114) indicated that MFT was achieved at 23.88 ± 1.80 °C, MVT at 38.13 ± 1.99 °C, and HTT at 49.56 ± 1.87 °C.

*H. fuscata* thermal responses assessed from the pronotum (*n* = 258) showed that the species had an MFT at 25.66 ± 2.06 °C, elevated its thermoregulation level to an MVT of 36.10 ± 1.46 °C, and became heat torpid at an HTT of 46.41 ± 2.58 °C. The responses to heat determined at the mesonotum (*n* = 197) exhibited MFT at 24.80 ± 1.89 °C, MVT at 36.39 ± 1.70 °C, and HTT at 48.56 ± 2.09 °C.

**Figure 2:**
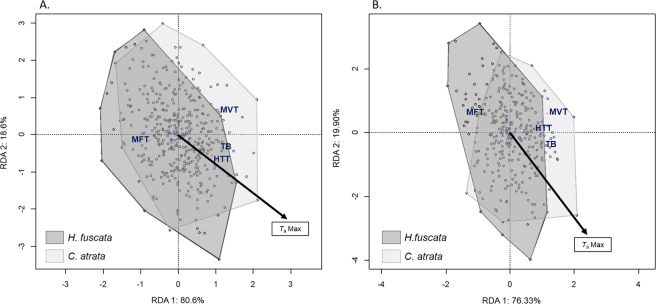
Ordination diagram of the two first axes of the RDA for comparative thermal responses for *C. atrata* and *H. fuscata* for the (**A**) pronotom and (**B**) mesonotum. MFT: minimum flighte temperature; MVT: maximum voluntary temperature; HTT: heat torpor temperature; *T*_*b*_Range: thermal breadth. This figure was generated using Vegan package on R Studio version 1.0.143.

In general, *C. atrata* tolerated heat better than *H. fuscata* in terms of MVT and HTT. The RDA models explained 11.77% and 13.17% of the total variation in temperature measurement from the pronotum and the mesonotum, respectively (Table 1). Both showed that thermal responses were significantly different between *C. atrata* and *H. fuscata*. Indeed, for both the pronotum and mesonotum temperature measurements, the species factor was the most significant (*p* = 0.001), representing the first axis for both ordination diagrams (Fig. 2A,B) and accounting for 80.6% and 76.33% of the inertia, respectively. Among four thermal responses, only MFT was greater for *H. fuscata*, whereas the others tended to be higher for *C. atrata* (Fig. 2A,B).

**Table 1:** Results of the RDA for comparative thermal responses measured from the pronotum and mesonotum of two cicada species. Percentage of inertia and *p*-values were calculated for each variable. The statistical significance of the entire model for each variable (marginal tests) was evaluated using Monte-Carlo permutation tests (n = 999).

	*Pronotum*		*Mesonotum*	
	% inertia	*p*-value	% inertia	*p*-value
***Full model***		**0.001**		**0.001**
Constrained	11.77		13.17	
Unconstrained	88.23		86.83	
***Variables effects***				
*T*_a_Max		**0.001**		**0.005**
Sex		0.252		**0.002**
Species		**0.001**		**0.001**

### Variation in morphological characters of each cicada species

Morphological measurements of 158 *C. atrata* individuals produced a total mass of 2.90 ± 0.47 g, a body length of 41.63 ± 1.62 cm, a mesothorax depth of 14.61 ± 0.63 cm, and a mesothorax width of 16.59 ± 0.75 cm. The result of the RDA showed that the effect of *T*_a_Max and sex did not significantly explain the morphological measurement matrix (Table 2).

**Table 2:** Results of the RDA conducted on morphological characteristics for *C. atrata* and *H. fuscata*. Percentage of inertia and *p*-values were calculated for each variable. The statistical significance of the entire model for each variable (marginal tests) was evaluated using Monte-Carlo permutation tests (n = 999). ns: not significant.

	*C. atrata*		*H. fuscata*	
	% inertia	p-value	% inertia	p-value
***Full model***		ns		**0.001**
Constrained			11.5	
Unconstrained			88.5	
***Variables effects***				
T_a_Max		ns		**0.002**
Sex		ns		**0.001**

**Figure 3:**
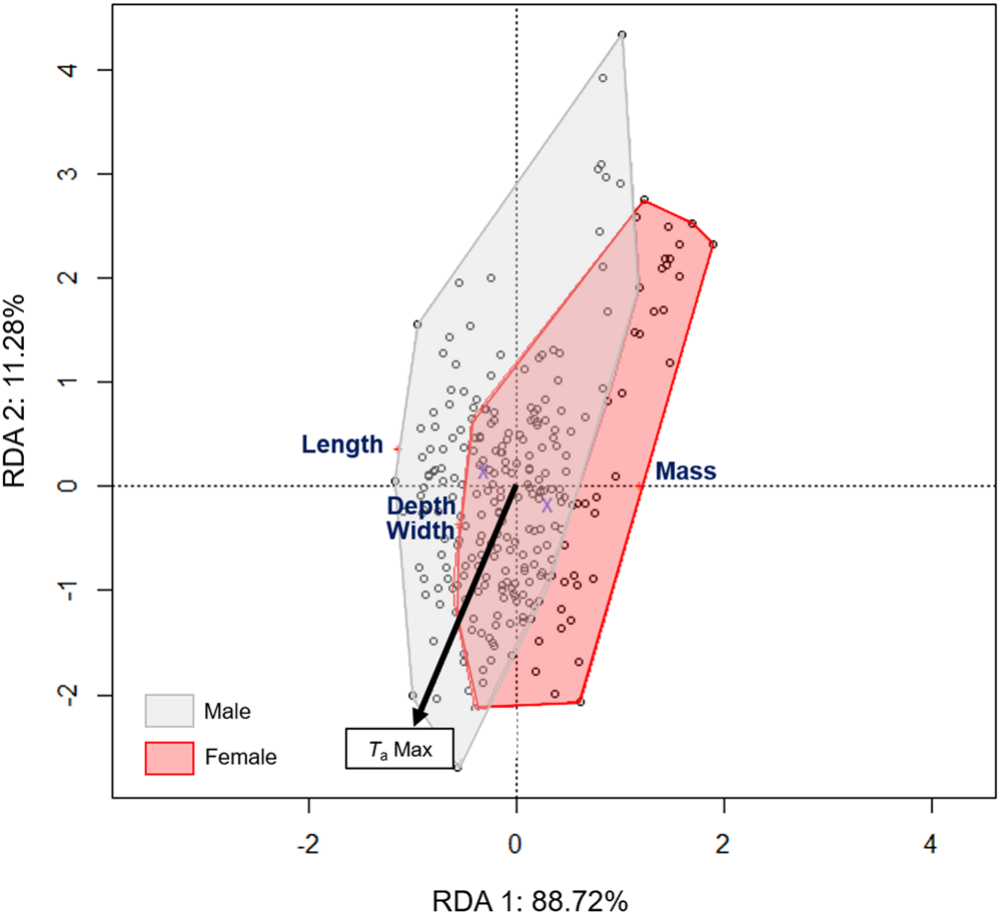
Ordination diagram of the two first axes of the RDA for morphologic characteristics of *H. fuscata*. The position of the variables are represented with convex hull based on sex. The part of the inertia explained by each axis is given. This figure was generated using Vegan package on R Studio version 1.0.143.

**Figure 4:**
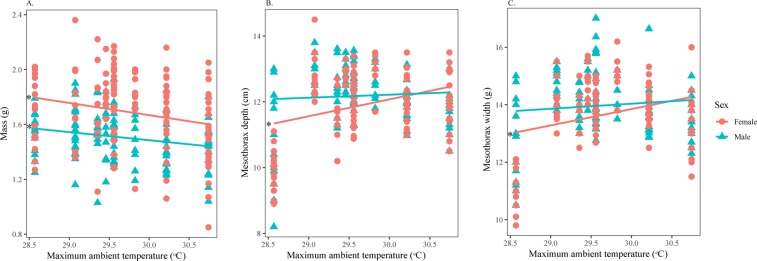
Variation in morphological characters of *H. fuscata* in response to *T*_a_Max divided by sex. Correlation between (**A**) Mass and *T*_a_Max, (**B**) Depth and *T*_a_Max, and (**C**) Width and *T*_a_Max. Asterisks denote significant linear regressions. This figure was generated using SPSS 22.

*H. fuscata* (*n* = 258) was characterized by a total live mass of 1.60 ± 0.25 g, with a body length of 32.54 ± 1.80 cm, a mesothorax depth of 12.06 ± 1.05 cm, and a mesothorax width of 13.84 ± 1.24 cm. The RDA model explained 11.5% of the recorded variation, in which sex was the principal factor (*p* = 0.001), represented along the first axis and accounting for 88.72% of the variance (Table 2, Fig. 3). *T*_a_Max was also significant (*p* = 0.002) and was represented along the second axis, accounting for 11.28% of the variance. Females were significantly heavier than males, but shorter in total body length (Supplementary Material 3). *H. fuscata* individuals exhibited a decrease in mass (Fig. 4A) but an increase in mesothorax size relative to *T*_a_Max (Fig. 4B,C). Linear regressions showed that *T*_a_Max influenced male and female morphological characteristics differently (Table 3); *T*_a_Max was significant for only the mass of males but not for other characteristics; on the contrary, *T*_a_Max was significant for almost all characteristics of females, except length.

**Table 3:** Linear regression analysis to assess the effect of *T*_a_Max on morphological characters of *H. fuscata* males and females.

Dependent variable	Independent variable	B	SE	*t*	P
**Male**					
Mass	Intercept	3.34	0.72	4.58	<0.001
	*T*_a_Max	−0.61	0.02	−2.49	**0.014**
Length	Intercept	29.18	6.27	4.65	<0.001
	*T*_a_Max	0.14	0.21	0.65	0.514
Depth	Intercept	8.95	4.06	2.21	0.029
	*T*_a_Max	0.11	0.14	0.80	0.427
Width	Intercept	8.05	4.77	1.69	0.094
	*T*_a_Max	0.20	0.16	1.24	0.216
**Female**					
Mass	Intercept	4.39	1.07	4.12	<0.001
	*T*_a_Max	−0.09	0.04	−2.54	**0.012**
Length	Intercept	24.02	6.84	3.51	0.001
	*T*_a_Max	0.26	0.23	1.14	0.258
Depth	Intercept	−4.35	4.05	−1.07	0.286
	*T*_a_Max	0.55	0.14	4.02	**<0.001**
Width	Intercept	−4.53	4.86	−0.93	0.354
	*T*_a_Max	0.61	0.16	3.75	**<0.001**

### Variation in thermal responses of each cicada species

Although *C. atrata* individuals exhibited some changes in their thermal responses, the species responded similarly to heat regardless of habitat conditions. The results of the RDA showed that both sex and *T*_a_Max had no significant effect on thermal responses obtained from the pronotum (*p* > 0.05, Table 4). For the mesonotum, sex was the only significant factor explaining the thermal responses (*p* = 0.029).

**Table 4:** Results of the RDA conducted on thermal responses for *C. atrata* and *H. fuscata*. Percentage of inertia and *p*-values were calculated for each variable. The statistical significance of the entire model for each variable (marginal tests) was evaluated using Monte-Carlo permutation tests (n = 999). ns: not significant.

	*C. atrata* – *Pronotum*		*C. atrata* – *Mesonotum*		*H. fuscata* – *Pronotum*		*H. fuscata* – *Mesonotum*	
	% inertia	*p*-value	% inertia	*p*-value	% inertia	*p*-value	% inertia	*p*-value
***Full model***		0.786		0.057		**0.001**		**0.001**
Constrained	ns		6.01		11.76		11.25	
Unconstrained	ns		95		88.24		88.75	
***Variables effects***								
T_a_Max		ns		0.269		**0.001**		**0.04**
Sex		ns		**0.029**		**0.011**		**0.001**
Width		ns		0.093		**0.011**		**0.001**

**Figure 5:**
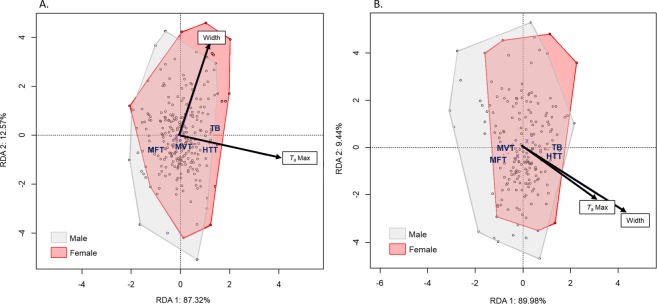
Ordination diagram of the two first axes of the RDA performed on MFT, MVT, HTT and *T*_*b*_Range measured at the (**A**) pronotum and (**B**) mesonotum separately for *H. fuscata*. The positions of the variables are represented with convex hulls based on sex. The part of the inertia explained by each axis is given. This figure was generated using Vegan package on R Studio version 1.0.143.

The RDA model showed that thermal responses from the pronotum were mostly influenced by *T*_a_Max (*p* = 0.001), followed by sex (*p* = 0.011) and width (*p* = 0.011) (Table 4). The model explained 11.76% of the total inertia, of which 87.32% was explained by axis 1 and 12.57% by axis 2 (Fig. 5A). Regarding the mesonotum, the thermal responses were mostly significantly influenced by sex (*p* = 0.001) and width (*p* = 0.001), followed by *T*_a_Max (*p* = 0.04) (Table 4, Fig. 5B). The thermal responses measured at the mesonotum were driven primarily by width rather than other factors (Supplementary Material 4).

### Intersexual variation in thermal responses of *H. fuscata*

**Figure 6:**
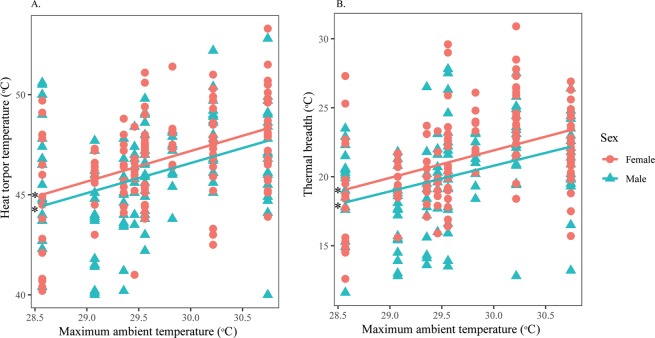
Intersexual variation in thermal responses of *H. fuscata* obtained from the pronotum according to *T*_a_Max. Correlation between (**A**) Heat torpor temperature and *T*_a_Max, (**B**) Thermal breadth and *T*_a_Max. Asterisks denote significant linear regressions. This figure was generated using SPSS 22.

**Figure 7:**
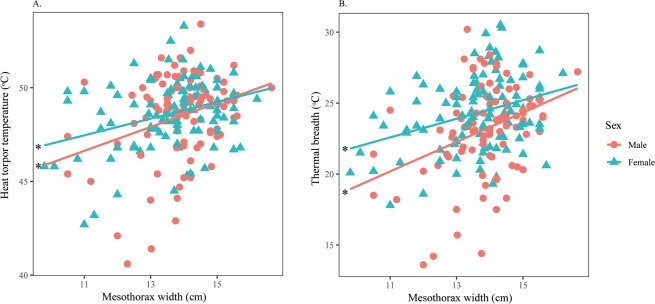
Relationship between thermal responses of *H. fuscata* obtained from the mesonotum and width. Correlation between (**A**) Heat torpor temperature and width, (**B**) Thermal breadth and width. Asterisks denote significant linear regressions. This figure was generated using SPSS 22.

For measurement from the pronotum, males and females had similar MFT and MVT. However, females tolerated heat significantly better (Fig. 6A) and had wider thermal ranges than males (Fig. 6B). Both sexes significantly increased their HTT and *T*_b_Range as *T*_a_Max increased (Supplementary Material 5), but no relationships were found between *T*_a_Max with either MFT or MVT (Table 5). Width displayed a significant negative effect on MFT of both sexes (Supplementary Material 6), while this factor was significantly positively correlated with HTT and *T*_b_Range of both males and females (Fig. 7).

**Table 5:** Linear regression analysis to assess the effect of *T*_a_Max and Width on thermal responses of males and females *H. fuscata* obtained from the pronotum.

Dependent variable	Independent variable	B	SE	*t*	P
**Male**					
MFT	Intercept	40.45	8.25	4.90	<0.001
	*T*_a_Max	−0.31	0.28	−1.10	0.272
	Width	−0.39	0.15	−2.59	**0.011**
MVT	Intercept	31.56	5.78	5.46	<0.001
	*T*_a_Max	0.20	0.19	1.02	0.310
	Width	−0.1	0.11	−0.94	0.351
HTT	Intercept	4.02	10.11	0.40	0.691
	*T*_a_Max	1.64	0.34	4.83	**<0.001**
	Width	−0.47	0.18	−2.54	**0.012**
TB	Intercept	−36.42	13.24	−2.75	0.007
	*T*_a_Max	1.94	0.44	4.38	**<0.001**
	Width	−0.08	0.24	−0.33	0.743
**Female**					
MFT	Intercept	38.17	7.81	4.89	<0.001
	*T*_a_Max	−0.27	0.28	−1.0	0.320
	Width	−0.33	0.14	−2.35	**0.020**
MVT	Intercept	28.98	5.94	4.88	<0.001
	*T*_a_Max	0.29	0.21	1.38	0.169
	Width	−0.11	0.11	−0.99	0.323
HTT	Intercept	0.49	8.52	0.06	0.954
	*T*_a_Max	1.45	0.30	4.83	**<0.001**
	Width	0.22	0.16	1.43	0.154
TB	Intercept	−37.68	11.95	−3.15	0.002
	*T*_a_Max	1.73	0.42	4.10	**<0.001**
	Width	0.56	0.22	2.55	**0.012**

## Discussion

In sum, our analyses suggest a local adaptation of thermal responses and thorax sizes in *H. fuscata* populations distributed along a thermal gradient in metropolitan Seoul, but not for the other cicada species. Although the results of our redundancy analysis showed high values of unconstrained variance (>85% in all of them), significant differences in thermal responses between two cicadas and within *H. fuscata* were determined. Specifically, thermal responses of *C. atrata* measured by MFT, MVT, HTT and *T*_*b*_Range indicated a better tolerance to heat stimuli than *H. fuscata*. Furthermore, no relationship was observed between the ambient temperature of the habitat and either morphology or thermal physiology of *C. atrata*. On the contrary, *H. fuscata* from habitats with higher ambient temperature had substantially enlarged thoraxes, endured heat better, and held wider fully active thermal ranges.

Although ambient temperature was significant for the pronotum’s HTTs and TBs of both sexes (Table 5), it was marginally significant for the mesonotum’s TB of males and HTT of females (Supplementary Material 3). To justify the effect of ambient temperature on thermal responses of *H. fuscata*, we conducted the RDA and linear regression again, using Mass instead of Width as a covariate (results not shown). RDA result shows that *T*_a_Max was significant for thermal responses of *H. fuscata* regardless of pronotum or mesonotum temperature. Furthermore, the result of linear regression analysis displays a consistent result between pronotum and mesonotum temperatures. We therefore conclude that overall *H. fuscata* increased their thermal tolerance in accordance to the increase in ambient temperature.

The increase in heat tolerance of *H. fuscata* resembles other research on urban-adapted insects, which indicates a close association between thermal tolerance and localized thermal clines^29,30,33^. Research on thermal responses of cicadas across a wide geographic range provides evidence that cicadas are more tolerant of warmer environments^41–43^. Cicadas of 38 species inhabiting Mediterranean habitats display an elevated HTT in accordance with the local thermal characteristics, regardless of taxonomic position or the diversity of particular plant species^50^. Our results further imply a localized thermal acclimatization of *H. fuscata*.

Here, we observed interspecific differences in thermal responses between *C. atrata* and *H. fuscata*, in which the warmer the habitat, the greater thermal responses *C. atrata* exhibited compared to the other species. This disparity can be explained by variation in species geographical origins: *C. atrata* originated in subtropical regions, whereas *H. fuscata* originated in tundra regions^35^. Additionally, segregation in microhabitat niches may contribute to how each species utilizes its habitat for thermoregulation. *C. atrata* perches mainly on top of the canopy, where it is exposed to solar radiation, whereas *H. fuscata* is found mainly on tree trunks in shaded environments^51^. As a result, adaptation to individual thermal regimes has led to variation in thermal responses between these species, a pattern that is well-discerned in other cicadas inhabiting tropical habitats^52^.

In line with other studies on thermal responses of cicadas, we found HTT depends strongly on the maximum environmental thermal regime^43,44,53^. This positive relationship was observed in *H. fuscata*, but not in *C. atrata*, regardless of habitat conditions. Such difference may be partially due to the origins of the two species. *C. atrata* is, therefore, more prone to experiencing higher thermal regimes and is adapted to high thermal conditions in metropolitan Seoul, thus exhibiting no difference in thermal tolerance across heterogeneous ambient temperatures. Environmental constraints applied to populations of the acorn ant, *Temnothorax curvispinosus*, are greater in lower latitudes, causing a reduction in evolutionary thermal responses relative to populations at higher latitudes^29^.

In contrast to *C. atrata*, distributions of *H. fuscata* at higher latitudes expose this species to colder environments, and the warmer conditions of metropolitan Seoul may induce a thermal acclimatization to warmer temperatures. Not only does urban warming seem to select for thermophilic species, but it also extends thermal tolerance ranges by elevating heat tolerance^30^. Here, as a function of ambient temperature, the thermal range of *H. fuscata* was extended toward warmer habitats. Better heat tolerance and wider thermal active ranges promote colonization of microhabitat niches generated by urbanization.

Besides thermal responses, our study suggest contrast relationships between thermal conditions and morphological characters of *H. fuscata* females, while thorax sizes increased as ambient temperature increased, total mass decreased. According to Bergman size clines, warmer environments usually trigger the growth rate of ectotherms. As a results, those from warmer environments tend to be bigger than the ones from colder environments^54^. The decrease of thorax sizes of females *H. fuscata* from warm to cooler habitats in this study shows support to Bergmann size clines. Besides, females cicadas developed at cooler environments were heavier than those at warmer environments. The flies examined by Crill *et al*.^55^ also exhibited similar changes in body dry mass to our study. Accordingly, only female flies developed at higher temperature were heavier in terms of dry mass whereas male flies were insensitive to their developmental temperature. However, the underlying mechanism is unclear. In our study, we observe such an inverse effect of temperature on thorax size and mass of females *H. fuscata*. The evolutionary explanation for this phenomenon remains elusive, thus we aim to elucidate it in further study. Besides, more investigations are needed to better understand this phenomenon and to clarify if these first results are linked to an exceptional population of small individuals in the coolest areas or if this could be a general trend.

Insect metabolic activity is accelerated under exposure to higher temperatures, which triggers an increase in body size^56^. Furthermore, warming climates have been proposed as causes of escalating growth rates of cicada nymphs underground, resulting in larger body sizes within a fixed development period^22^. Females of the scale insect *Melanasimpis tenebricosa* inhabiting warmer tree canopies exhibit larger body size than those living in cooler tree canopies^57^. An increase of 2.5 °C in rearing temperature promotes the development of larger females in the southern green stink bug, *Nezara viridula*^58^. There is also evidence that larger body size may be driven by local adaptation to warmer habitats. In periodical cicadas, for instance, the more southerly diverged *M. tredecim* experiences higher temperatures as adults possess larger bodies^22^. Larger bodies provide multiple ecological and evolutionary advantages by contributing positively to survival, fecundity, and mating success^59^. As a result, a larger size could result from selection for greater fecundity in female cicadas^23^.

The relationship between body size and thermal tolerance has been explored in various terrestrial insects. Although there are cases where superior heat tolerance is found in smaller^60,61^ or intermediate-sized insects^62^, larger species tend to tolerate higher temperatures better than smaller species do^57,58,62,63^. In our study, larger *H. fuscata* individuals had higher heat tolerance. Consequently, better thermal tolerance capacity is suggested to provide higher fitness to female cicadas^3^. Although body size of male *H. fuscata* was not correlated with ambient temperature, it was found to be associated with acoustic properties of cicada songs^64–66^, which act as premating signals to attract conspecific mates. Furthermore, songs of warmer males are higher in intensity and are able to travel further in the air^67^, increasing the transmission of their mating signals to prospective mates.

## Conclusion

Our thermal tolerance experiments indicate a local adaptation of thermal responses and thorax sizes along a thermal gradient of *H. fuscata* in metropolitan Seoul. Whether such variation in heat tolerance is caused by phenotypic plasticity or evolutionary adaptation to environmental conditions is unclear. However, acclimatization to anthropogenic perturbation as a consequence of urbanization may be partially responsible. This is the first study to notice variation in thermal tolerance of cicadas at the local urban scale. Our research highlights the importance of taking localized thermal regimes into consideration when examining species-specific responses to escalating urban warming caused by urbanization.

## Supplementary information

Supplementary Information.

## Data Availability

The datasets analysed during the current study are available in the Mendeley Data repository, DOI: http://dx.doi.org/10.17632/4z4n7p5gxt.2#file-ae6d6c15-bf0a-4fde-8f32-4f62ebf8f96c.
