# Supplementary material for: Not all cicadas increase thermal tolerance in response to a temperature gradient in metropolitan Seoul
Source: Sci Rep. 2020 Jan 28;10:1343. doi: 10.1038/s41598-020-58276-0 (PMC6987172; doi:10.1038/s41598-020-58276-0)
Supplement: Supplementary file 1 — Supplementary Information. [file 41598_2020_58276_MOESM1_ESM.pdf]

Supplementary material 1. One-way analysis of variance to test the difference in isothermal, wetness, greenness and imperviousness among nine sampling areas distributed within and surrounding metropolitan Seoul.

		**Sum of Squares**	***df***	**Mean Square**	**F**	***P***
Isothermal	Between groups	0.141	8	0.018	965.283	< 0.001
	Within groups	< 0.001	18	< 0.001		
	Total	0.142	26			
Wetness	Between groups	0.006	8	0.001	0.773	0.631
	Within groups	0.017	18	0.001		
	Total	0.023	26			
Greenness	Between groups	0.032	8	0.004	2.509	0.050
	Within groups	0.029	18	0.002		
	Total	0.061	26			
Imperviousness	Between groups	0.092	8	0.012	1.316	0.297
	Within groups	0.158	18	0.009		
	Total	0.250	26			

Supplementary material 2. List of sampling areas and the sample size of each species collected at each area. Minimum and maximum temperatures refer to the average minimum and maximum ambient temperatures in summer from 2010 to 2015, respectively.

**Category**	**Location**	**Latitude**	**Longitude**	**Minimum temperature (ºC)**	**Maximum temperature (ºC)**	***C. atrata***		***H. fuscata***	
						**Male (n)**	**Female (n)**	**Male (n)**	**Female (n)**
Hottest temperature	Seocho	37.5021° N	127.0205° E	22.3	30.7	12	29	20	27
	Gwangjin	37.5359° N	127.0714° E	22.5	30.2	21	9	18	22
	Yeongdeungpo	37.5340° N	126.9058° E	22.6	29.8	12	20	11	9
	Gwangmyeong	37.477° N	126.8736° E	22.0	29.6	0	0	21	22
	Namyangju	37.6583° N	127.1455° E	20.0	29.5	0	0	10	10
	Nowon	37.6308° N	127.0669° E	20.3	29.4	4	14	17	7
	Soha	37.4550° N	126.8853° E	20.9	29.3	21	8	0	0
	Jookyo	37.6632° N	126.8392° E	21.6	29.1	2	3	20	11
Coolest temperatures	Gwanak	37.4833° N	126.9105° E	20.2	28.6	1	2	17	16

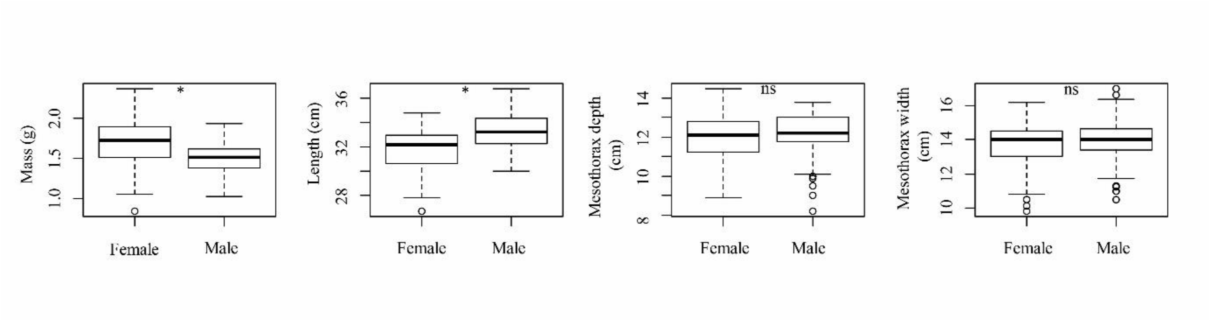

Supplementary material 3. Intersexual differences in morphological characters of *H. fuscata*. A *t*-test was performed for mass, and Kruskal-Wallis tests were performed for length, depth and width. Asterisks denote significant differences, ns: not significant.

Supplementary material 4. Linear regression analysis to assess the effect of T_aMax and Width on thermal responses of males and females *H. fuscata* obtained from the mesonotum.

**Dependent variable**	**Independent variable**	***B***	***SE***	***t***	***P***
Male					
MFT	Intercept	43.70	8.81	4.96	< 0.001
	T_aMax	-0.44	0.29	-1.51	0.134
	Width	-0.38	0.16	-2.30	**0.023**
MVT	Intercept	44.96	8.60	5.23	< 0.001
	T_aMax	-0.13	0.29	-0.45	0.651
	Width	-0.32	0.16	-1.97	0.052
HTT	Intercept	23.66	11.37	2.08	0.040
	T_aMax	0.55	0.38	1.45	0.150
	Width	0.614	0.211	2.90	**0.005**
TB	Intercept	-20.04	15.15	-1.32	0.189
	T_aMax	1.0	0.51	1.97	0.052
	Width	0.99	0.28	3.52	**0.001**
Female					
MFT	Intercept	28.90	8.73	3.31	0.001
	T_aMax	-0.06	0.30	-0.20	0.841
	Width	-0.20	0.15	-1.36	0.176
MVT	Intercept	36.42	7.64	0.30	< 0.001

	T_aMax	0.08	0.27	0.30	0.768
	Width	-0.20	0.13	-1.51	0.134
HTT	Intercept	27.14	8.01	3.39	0.001
	T_aMax	0.55	0.28	1.97	0.051
	Width	0.37	0.14	2.75	**0.007**
TB	Intercept	-1.76	11.48	-0.15	0.879
	T_aMax	0.61	0.40	1.53	0.129
	Width	0.58	0.19	2.96	**0.004**

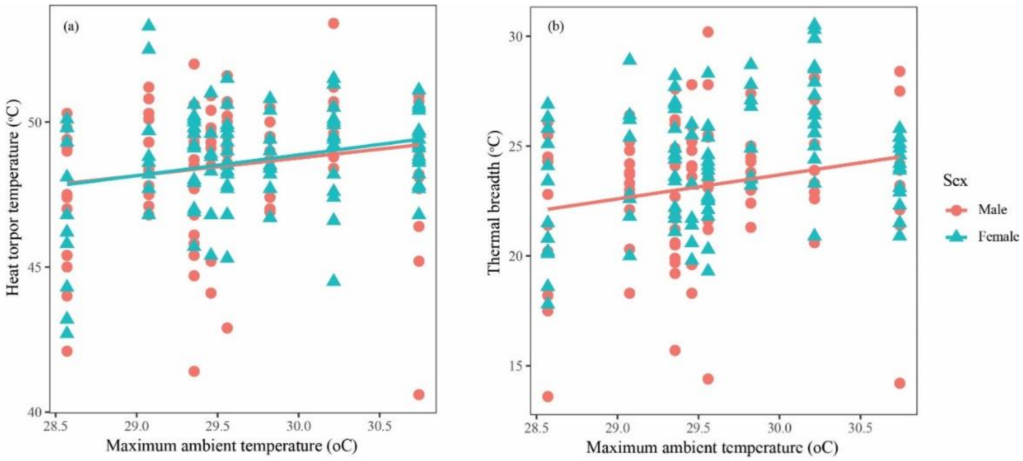

Supplementary material 5. Intersexual variation in thermal responses of *H. fuscata* obtained from the mesonotum according to T_aMax. Correlation between (a) heat torpor temperature and T_aMax, and (b) thermal breadth and T_aMax.

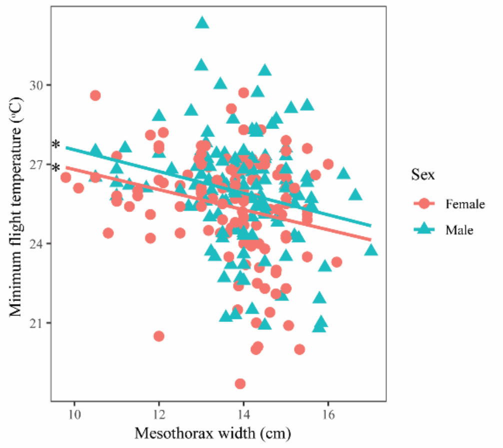

Supplementary material 6. Relationship between thermal responses of *H. fuscata* obtained from the mesonotum and Width. Arterisks denote significant linear regressions.
